# Inferring models of opinion dynamics from aggregated jury data

**DOI:** 10.1371/journal.pone.0218312

**Published:** 2019-07-01

**Authors:** Keith Burghardt, William Rand, Michelle Girvan

**Affiliations:** 1 Information Sciences Institute, University of Southern California, Marina del Rey, CA, United States of America; 2 Poole College of Management, North Carolina State University, Raleigh, North Carolina, United States of America; 3 Dept. Of Physics, University Of Maryland, College Park, Maryland, United States of America; 4 Institute for Physical Science and Technology, University Of Maryland, College Park, Maryland, United States of America; 5 Santa Fe Institute, Santa Fe, New Mexico, United States of America; Northwestern University, UNITED STATES

## Abstract

Jury deliberations provide a quintessential example of collective decision-making, but few studies have probed the available data to explore how juries reach verdicts. We examine how features of jury dynamics can be better understood from the joint distribution of final votes and deliberation time. To do this, we fit several different decision-making models to jury datasets from different places and times. In our best-fit model, jurors influence each other and have an increasing tendency to stick to their opinion of the defendant’s guilt or innocence. We also show that this model can explain spikes in mean deliberation times when juries are hung, sub-linear scaling between mean deliberation times and trial duration, and unexpected final vote and deliberation time distributions. Our findings suggest that both stubbornness and herding play an important role in collective decision-making, providing a nuanced insight into how juries reach verdicts, and more generally, how group decisions emerge.

## Introduction

What mechanisms underlie collective decision-making? Recent research on collective decision making has compared statistical patterns in empirical data to models [1–5] and tested how opinions change in controlled experimental settings [6–10]. Although both methods have provided substantial insight into the dynamics of collective decisions, the mechanism underlying how groups make decisions that do not end in unanimous agreement is underexplored. In addition, it is often difficult to determine through available data whether opinions form independently or if influence plays a role because either mechanism generally displays similar patterns in data [11, 12]. Despite this difficulty, we ask whether clues in data can hint at the role influence (or “herding” [13]) might play in group decision-making. We expect that opinions shift due to influence, but methods to test this intuition is lacking. Our recent work modeling voting behavior suggests that both herding and “increasing stubbornness”, in which individuals increasingly hold onto their opinion the longer they have it, help to explain data on vote distributions [5]. Do related models for other datasets reach similar conclusions? We explore these questions by comparing data of collective decision making in which decisions are made in the absence of complete consensus to a battery of plausible models with and without influence and/or stubbornness.

We use data on jury deliberation as a case study in this paper. Over one million Americans are impaneled for jury duty each year, making juries a common aspect of the modern-day justice system [14]. Juries decide everything from the guilt of a criminal defendant to awarding damages to a plaintiff in a civil case. Our work complements previous research in which judicial rulings were affected by factors unrelated to the specific cases [15]. By analyzing jury data in bulk, we aim to understand how the mechanisms of jury opinion formation that are not directly related to the facts of the case couple with the laws defining hung juries (situations in which jury opinions are considered too divided to reach a verdict) to shape decision-making patterns observed in data. Namely, we model juror decisions by fitting competing microdynamic models to data on aggregate distributions. It may seem counter-intuitive to match a model of how opinions change dynamically to data that is static. Recent work, however, has shown that different dynamical models of group opinion formation create different distributions in the amount of time it takes for groups to reach consensus [16, 17], which inspires us to reverse engineer a minimal model that provides a compelling match to the distributions. Furthermore, by matching the joint distribution instead of either distribution alone strongly limits the possible dynamical models that can explain the data. For example, in contrast to many models of group decision-making [16, 18–20], juries rarely reach complete agreement before they stop deliberating. It may therefore be possible to match the deliberation time alone with unrealistic models in which all jurors reach agreement, but the joint distribution rules out such models.

There has been a long history of assuming jurors act independently, going back to Condorcet’s Jury Theorem, which helped motivate trials by juries [21]. This and similar research found that independence allows for groups, such as juries, to create better decisions than single individuals (jurors) [21–24]. Intrinsic in our paper’s analysis is to test whether independent jurors are an appropriate assumption for a model, and if they are not, what sort of influence mechanism may exist. The effect of influence on jurors is uncertain. Psychological research suggests that social signals, represented as “descriptive norms”, allow individuals to efficiently determine what is a good idea [25], and popularity may be representative of quality in some cases [26]. Moreover, sometimes deliberation produces more accurate guesses [8, 9]. However, some research shows that while interactions of decentralized groups produce better decisions, groups centralized around one person can produce poorer decisions [10]. Further, significant psychological research suggests group decisions can be detrimental [27]. This includes evidence that minority viewpoints are discounted even when they are correct [28], and that individuals avoid speaking when they disagree with the majority [29] (similar to groupthink [30]). Both of these effects reinforce the majority opinion, as in majority influence [31, 32] and conformity [33–35], meaning a poorer idea may become popular over better minority ideas. A recent theoretical model also suggests that correlated juror decisions (e.g., from influence) can undermine their collective accuracy [36]. Overall, influence may or may not be beneficial, and different mechanisms of influence, e.g., a centralized or decentralized network, may be a significant factor in the success of deliberation. By creating a mechanistic model consistent with data, we hope to provide insights into the role of influence in jury deliberation.

### Features of the data

Jury deliberation is an ideal test bed for models of opinion dynamics. Jurors are exposed to the same information during the trial, are instructed not to discuss the trial with non-jurors, and cannot learn about the trial from outside sources [37–41], therefore opinion variation between jurors is likely due to internal factors, such as influence instead of common external factors, such as varying levels of information.

**Fig 1:**
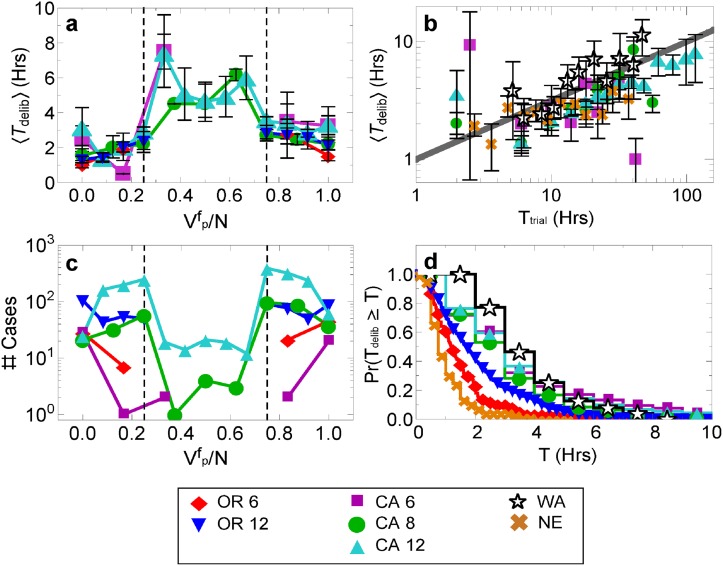
Data summary. (a) The mean deliberation time versus the fraction of jurors voting for the plaintiff. (b) The mean deliberation time versus the trial time across several datasets. (c) The distribution of the fraction of jurors voting for the plaintiff in the final vote, $V_{\text{p}}^{\text{f}}/N$, and (d) the complementary cumulative distribution of deliberation. Error bars represent 90% confidence intervals in the mean.

The purpose of our modeling efforts is to explain four features of the data shown in Fig 1:

1. Mean deliberation time, 〈*T*_delib_〉 as a function of the fraction of jurors voting for the plaintiff, $\left(V_{\text{p}}^{\text{f}}/N\right)$ (Fig 1a)
2. 〈*T*_delib_〉 scales as the square root of the trial time, (*T*_trial_)^1/2^ (Fig 1b)
3. Near absence of trials in which juries are hung (Fig 1c)
4. Left-skewed distribution of deliberation time (Fig 1d)

See Table 1 for descriptions of variables we use throughout the paper. In more detail, we first aim to model why the mean deliberation time, 〈*T*_delib_〉, is lowest when the fraction of jurors voting for the plaintiff in the final vote, $V_{\text{p}}^{\text{f}}/N$ is 0 or 1, and highest when $V_{\text{p}}^{\text{f}}/N\approx 0.3$ or $V_{\text{p}}^{\text{f}}/N\approx 0.6$ (Fig 1a). Notably, when $0.25<V_{\text{p}}^{\text{f}}/N<0.75$ juries are considered “hung” (they are dismissed and a new trial is given to the defendant) for the OR and CA civil trials [42, 43]. It is therefore not a coincidence that the deliberation time is highest when $0.25<V_{\text{p}}^{\text{f}}/N<0.75$: juries try to avoid hanging if they can. Hanging is externally given; once juries are unable to reach the required majority, they discuss this to the judge who may tell them to deliberate further. If they remain deadlocked, the judge dismisses them and the defendant is usually given a retrial. This deliberate effort to avoid hanging also helps explain why hanging is so rare, as seen in Fig 1c. Perhaps not surprisingly, however, the threat of a jury hanging does not imply that juries usually reach complete agreement; instead there is often one or more jurors in disagreement. We also observe that 〈*T*_delib_〉 scales with the trial time, *T*_trial_, as 〈*T*_delib_〉 ∼ (*T*_trial_)^1/2^ (Fig 1b), where trial time is defined as the total time a jury is in trial, usually before deliberating. We might expect a correspondence between trial time and deliberation time, because a longer trial time may imply juries have to argue over more facts. That said, the power-law relation across several different datasets is unexpected and hints at a common underlying process. Importantly, this scaling law is not strongly correlated with the final vote (Fig G in the S1 File), even though both affect the mean deliberation time. Furthermore, in Fig 1d, we notice that deliberation time distribution is highly left-skewed. We finally mention that 〈*T*_delib_〉 does not depend strongly on jury size (Fig F in the S1 File). Some common opinion models show a strong dependence on the size of the deliberating group [20, 44, 45], so we can rule these out as candidate models of the data.

**Table 1:** Variable descriptions.

Variable Description	
〈*T*_delib_〉	Mean deliberation time
$V_{\text{p}}^{\text{f}}/N$	Fraction of jurors voting for the plaintiff
*T*_trial_	Trial time

In this paper, we model jury datasets for civil trials in Oregon (OR) [42] and California (CA) [46]. We find qualitatively similar behavior in the Washington (WA) and Nebraska (NE) datasets whose data is less complete [47, 48]. Namely, the WA and NE data shows the deliberation time scaling behavior 〈*T*_delib_〉∼(*T*_trial_)^1/2^ (Fig 1b) but also the left-skewed deliberation time distribution (Fig 1d). We also find qualitatively similar behavior for criminal trials in the OR dataset (see Fig C in S1 File). Criminal cases have a similar function, $\langle T_{\text{delib}}\rangle \left(V_{\text{p}}^{\text{f}}/N\right)$, as well as a lack of hung juries and a similarly left-skewed deliberation time distribution. We fit empirical joint distributions of final votes and deliberation times to model distributions through maximum likelihood estimation of model parameters, therefore we can better understand opinion dynamics despite lacking access to time-series data.

Just like any model, we do not claim our best-fit model is the only model that can fit the data. It is instead a minimal model that allows us to begin understanding the true deliberation dynamics. A major limitation in the data, however, is that no two trials are exactly the same, therefore aggregating over heterogeneous trials may strongly affect our results [49]. To test for this effect, we split data into more homogenous groups with the same *N* and similar *T*_trial_, because *N* affects $V_{\text{p}}^{\text{f}}$ and *T*_trial_ affects *T*_delib_. We find, however, that splitting the data does not affect the qualitative behavior of $V_{\text{p}}^{\text{f}}$, and *T*_delib_. As seen in Fig 1, the qualitative findings are not affected by the various datasets and various numbers of jurors deliberating. We will also show that splitting data by trial time similarly changes some quantities, but leaves the qualitative results unaffected. These results so far suggest that heterogeneity should not significantly affects our findings.

## Methods and materials

### Data gathered

The jury data we study is taken from Multnomah County, Oregon (OR) [42], San Francisco County, California (CA) [46], Thurston County, Washington (WA), and Douglas County, Nebraska (NE) [47, 48]. See Table 2 for a summary of data. In the CA and OR datasets, the deliberation time and final vote are known, but the OR dataset does not record *T*_trial_. The WA and NE does not record the final vote of jurors. Before cleaning, we have 1162, 6482, 151, and 156 datapoint for the OR, CA, WA, and NE datasets respectively. After cleaning, we have 1158, 2117, 151, and 135 data points for the OR, CA, WA, and NE datasets respectively (see SI for details on how data is cleaned). Relatively little data was removed except for the CA data, mostly becuase we had stricter conditions for data completeness, and only consider trials where jurors deliberate on one count, to simplify the condition we are modeling. In the OR dataset, some trials are criminal trials, which have different rules about when juries are hung (see Fig C & Table C in the S1 File) [50], therefore we focus on civil trials in the main text. In the CA dataset, all trials were civil trials. In the WA and NE datasets, on the other hand, the final vote was not recorded, therefore we did not attempt to model the dynamics. This study was approved by the UC Davis IRB board, where K.B. was a researcher at the time (IRB ID: 1180117-1). No consent was obtained because the data was analyzed anonymously after the fact.

**Table 2:** Data summary.

Data	Trial Time	Final Vote	Num. Trials (Before Cleaning)	Num. Trials (Cleaned)	Num. Civil Trials
OR	No	Yes	1162	1158	661
CA	Yes	Yes	6482	2117	2117
WA	Yes	No	151	141	-
NE	Yes	No	156	135	-

### Data modeling

#### Influence with stubbornness model

In order to explore the types of processes that could generate the data, we propose an influence model with increasing stubbornness. Our motivation for an influence-based model relates to the multitude of studies of conformity or influence in small groups [30–32, 34, 35, 51]. Within the large space of plausible models, we focus on a simple model inspired by majority influence [31, 32]. Majority influence is the notion that people will tend to comply with the majority, but we do not try to distinguish people choosing the majority opinion as a form of compliance, or internalization [52]. Due to data limitations, we cannot rule out alternative models, for example some cases could be near consensus because cases are clearly in favor of the defendant or plaintiff. We therefore focus on these models as reasonable examples of plausible models. We consider a plausible model with a small set of parameters, and then check whether any of these parameters could be removed without affecting the quality of the fit.

**Fig 2:**
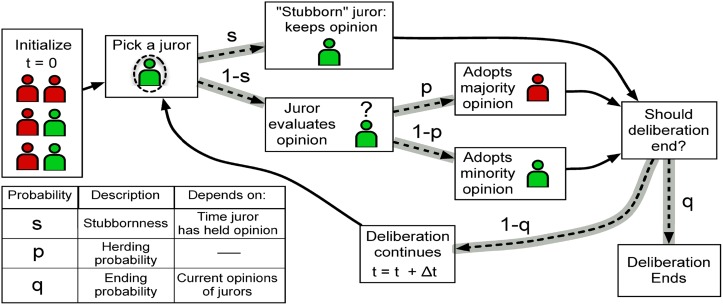
Schematic of the influence with stubbornness model. Solid lines correspond to deterministic transitions, while dashed lines correspond to probabilistic transitions. Jurors are first initialized to have one of two opinions (for the plaintiff or defendant). At each timestep, a random juror is picked and considers re-evaluating their opinion with probability 1 − *s*, where *s* is “stubbornness”. If they do re-evaluate, they pick the majority opinion with probability *p*, and the minority with probability 1 − *p*. At the end of each timestep, the jury stops deliberating with probability *q*. See (1), (2), and (3) for definitions of *s* and *q*.

In our model, the initial condition is that each juror votes for the plaintiff with a probability *b*. The value of *b* is chosen such that the number of juries whose majority opinion is for the plaintiff matches the empirical data. Because majority opinion tends to become the dominant opinion in our model, this allows us to match the asymmetry in the number of jurors with verdicts for the plaintiff or the defendant to the data. Once the simulation starts, the modeled jurors tend to adopt the majority opinion and juries end deliberation at a rate that depends on the current vote (number of jurors currently leaning for the plaintiff and for the defendant). The former incorporates a simple mechanism for juror majority influence that enables the supermajorities observed in data, while the latter allows for deliberation times to vary for different non-consensus opinions. In addition, we add a stubbornness property, in which jurors increasingly hold on to their current opinion. This helps facilitate the strong non-consensus patterns from data. More specifically, as shown in Fig 2, at each timestep in the model a random juror is selected and considers re-evaluating their current opinion with probability 1 − *s*, where *s* reflects their stubbornness and depends on how long they’ve held their current opinion. A timestep, Δ*t*, is chosen to be 1 minute. Simulations with significantly smaller or larger timesteps (between 15 seconds and 4 minutes) do not show consistently better fits (for most data, p-value> 0.1 using the likelihood ratio test [53]). If the juror decides to reevaluate, they pick the majority opinion with probability *p*, and the minority with probability 1 − *p*. This rule is based on the Majority Voter Model [16, 54, 55], therefore our influence with stubbornness model can be thought of as an extension of this model. At the end of each timestep, the jury stops deliberating with probability *q*, which depends on the current set of juror opinions. The stubbornness probability, *s*, depends both on how long the juror has held their current opinion and whether the current set of opinions meet the hung condition:

$$s\left(V_{\text{p}}\left(t\right)\right)=\sum_{i=0}^{\tau /\Delta t}\mu_{\text{eff}}\left(V_{\text{p},\text{i}}\right)\Delta t,$$ (1)

where *t*_0_ is the time a juror adopted its most recent opinion, *τ* is the time a juror has held their current opinion, and *μ*_eff_ is the rate jurors become more stubborn:

$$\mu_{\text{eff}}\left(V_{\text{p},\text{i}}\right)=\{\begin{array}{cc} \mu & \frac{V_{\text{p},\text{i}}}{N}\leq 1-\phi ,\;\frac{V_{\text{p},\text{i}}}{N}\geq \phi \\ f\mu & 1-\phi <\frac{V_{\text{p},\text{i}}}{N}<\phi \end{array},$$ (2)

where *f* is the reduction in this rate when juries are hung at time *t* = *t*_0_ + *i*Δ*t*. *V*_p,i_ divided by *N* is the vote of the jury at *t* = *t*_0_ + *i*Δ*t*. In other words, *s* depends on how juries vote in the past via the *μ*_eff_ vote-dependent parameter. If the stubbornness probability, *s*, were instead set to a constant, that would only have the general affect of changing the timescale of the dynamics. We incorporate *increasing* stubbornness (*s* grows with *τ*) as a behavioral hypothesis. The jury’s tendency to reach a non-hung decision is captured by making the stubbornness rate *μ*_eff_ lower under hung conditions, meaning that jurors do not hold onto their opinions as strongly as they would otherwise, presumably to lessen the probability that the jury hangs.

At the end of each timestep, the probability for a jury to stop deliberating, *q*, is determined:

$$q\left(V_{\text{p}}\left(t\right)\right)=\{\begin{array}{cc} q_{0}+\alpha \left|\frac{V_{\text{p}}\left(t\right)}{N}-1/2\right| & \frac{V_{\text{p}}\left(t\right)}{N}\leq 1-\phi ,\;\frac{V_{\text{p}}\left(t\right)}{N}\geq \phi \\ q_{0} & 1-\phi <\frac{V_{\text{p}}\left(t\right)}{N}<\phi \end{array},$$ (3)

where *q*_0_ = 0.3*α* is the base rate of quitting, and $\alpha |\frac{V_{\text{p}}\left(t\right)}{N}-1/2|$ is expected to be greater than 0 because the jury is more likely to stop deliberating if it is not currently hung. We have *q*_0_ > 0 because otherwise juries would never stop deliberating when juries are completely hung. Varying this value between $0.1\hat{\alpha }-1.0\hat{\alpha }$, however, produces statistically equivalent fits (p-values > 0.1). If a jury stops deliberating at time *t*, then the time and the final vote, $V_{\text{p}}^{\text{f}}=V_{\text{p}}\left(T_{\text{delib}}=t\right)$, are recorded.

To summarize, the influence with stubbornness model, as shown in Fig 2, involves three different transition probabilities: *p*, *q*, and *s*. These transition probabilities are constructed from a total of four fitting parameters: *μ*, *α*, *f*, and *p*, (described in Table 3) and three fixed parameters: Δ*t*, the length of a time step; and *q*_0_, the base rate of quitting, and *b*, the bias of the initial opinion (described in Table 4).

**Table 3:** Tuned model parameters.

Parameter Descriptions		Range
*p*	Herding probability	0 − 1
*μ*	Stubbornness rate	≥ 0
*f*	Reduces *μ* when hung	0 − 1
*α*	Controls rate of ending deliberation	≥ 0

**Table 4:** Fixed model parameters.

Fixed Parameter Descriptions		Value
*b*	Controls initial opinion preference	*b* such that the fraction of juries that are majority for-the-plaintiff matches data
Δ*t*	Timestep of simulations	1 minute
*q*_0_	Controls rate of ending deliberation when hung	0.3*α*

#### Model fitting

To find $\hat{p}$, $\hat{\alpha }$, $\hat{\mu }$, and $\hat{f}$, both in the full model, and models with different parameters removed, we use maximum likelihood estimation via grid search. Some events exist in the data which had a near-zero probability of occurring in the model. Because the log-likelihood would otherwise be undefined, we added a small base probability of between 10^−4^ and 10^−14^ to the models with no significant qualitative changes in the results (all values in the paper have a base probability of 10^−11^). Finally, the distributions we used to fit the influence with stubbornness model variants to the data were created from 1.6 × 10^5^ simulations per parameter value. There was an inherent limit in the probability resolution (6 × 10^−6^), but we do not believe this significantly affects our results. All mean values and parameter confidence intervals come from bootstrapping and fitting the data 10^4^ times.

## Results

### Influence with stubbornness model

Fig 4 shows that not only can the influence with stubbornness model explain vote and time distributions, but it can also explain the peaks in deliberation time near the critical fraction of voters $V_{\text{p}}^{\text{f}}/N\approx 0.3$ and 0.6. This appears to be due to important factors included in the influence with stubbornness model: the instability of juries having 50/50 split decisions, and the ability for juries to stop deliberating even then they have not reached complete consensus. We create a simplified model to explain the peak seen in the simulation in the Supporting Information. See Fig A in the S1 File for a simple explanation of the simplified model we use. We should caution, however, that the model’s assumption that each jury contains homogenous jurors is a simplification, which follows assumptions made in previous models [1, 5]; real juries likely have jurors who exhibit a variety of behaviors.

### Reduced models

**Fig 3:**
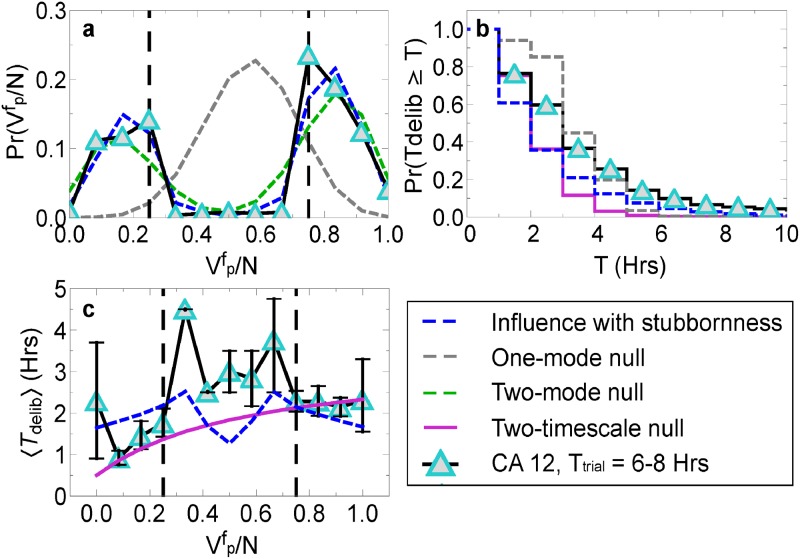
Comparison of models. Normalized log-likelihoods for the null models and the influence with stubbornness model variants. Models above -1 explain the data better than the influence with stubbornness model, while those below -1 perform worse. (a) The relative fit of the one-mode, two-mode, and two-timescale null models, along with “no herding” model, in which *p* = 0.5, “no stubbornness” model, in which *μ* = 0, “no vote dependence” model, in which the model dynamics do not depend on the number of jurors voting for the plaintiff, and the “no hung conditions” model, in which jury dynamics do not depend on whether the jury is currently hung. (b) In a zoomed-in graph, the influence with stubbornness model variants seen in (a) perform worse than the full model.

We construct variants of the full model in order to identify which mechanisms are most important for capturing the observed patterns. First, we test whether herding affects jury trials by setting *p* = 0.5 (Fig 3 in the main text and Fig C in the S1 File). If *p* = 0.5, a juror would have equal preference to pick the majority opinion as the minority one, therefore we remove herding from our model. We see that the fit is significantly worse, therefore herding appears to affect the outcomes of jury trials. We next test the role of increasing stubbornness by setting *μ*_eff_ = 0. This is equivalent to removing juror stubbornness. Removing the increasing stubbornness parameter, however, produces significantly poorer fits to the data (Fig 3 in the main text and Fig C in the S1 File). A similar conclusion is reached in previous work that matches a model to election data in several European countries [5]. Because highly disparate datasets have similar conclusions about the importance of increasing stubbornness, we believe it plays a fundamental role in opinion dynamics. Setting the stubbornness probability, *s*, to a constant greater than 0 should only generally decrease the timescale of the dynamics, presumably making the final vote distribution more similar to the initial vote distribution, therefore in the interest of space, we leave out further model variants of this type. Finally, to better understand how the hung conditions affect jury behavior, we fit a model with no dependence on hanging: *μ*_eff_ = *μ* and *q*(*t*) = *q*_0_ + *α*|*V*_p_(*t*)/*N* − 1/2|. In this “no hung conditions” model, jurors would presumably act the same whether or not the jury was hung. Neither the stubbornness rate, nor the quitting rate, depends on whether the jury is currently hung. The probability for the jury to end deliberations, however, still increases linearly with the amount of consensus among jurors. Similarly, to test the importance of the current vote has on jury dynamics, we create a “no vote dependence” variant in which *μ*_eff_ = *μ*, and *q* is a fitted constant. The jury therefore acts the same regardless of the current vote. Both of these variants show poorer agreement to the data compared to the full model (Fig 3 in the main text and Fig D in the S1 File). We finally tested removal of the hung conditions from either the stubbornness rate (Eq 2) or the stopping probability (Eq 3), but not both. We find that removing the hanging dependence of the stubbornness rate fits the data worse than removing the hanging dependence of the stopping probability (Figs B & E in the S1 File). Hanging may therefore affect how juror opinions change more than it affects how juries decide to end deliberations. In summary, the full model agrees with data significantly better than model variants that remove herding, stubbornness, hung-conditions, and vote-dependent behavior.

### Null models

Although we created a model that matches many qualitative features of the data, there are potentially other models that may fit the data better. One important assumption in our model, for example, is that jurors influence each other. Are there simple models that can explain the properties we see that do not depend on influence?

**Fig 4:**
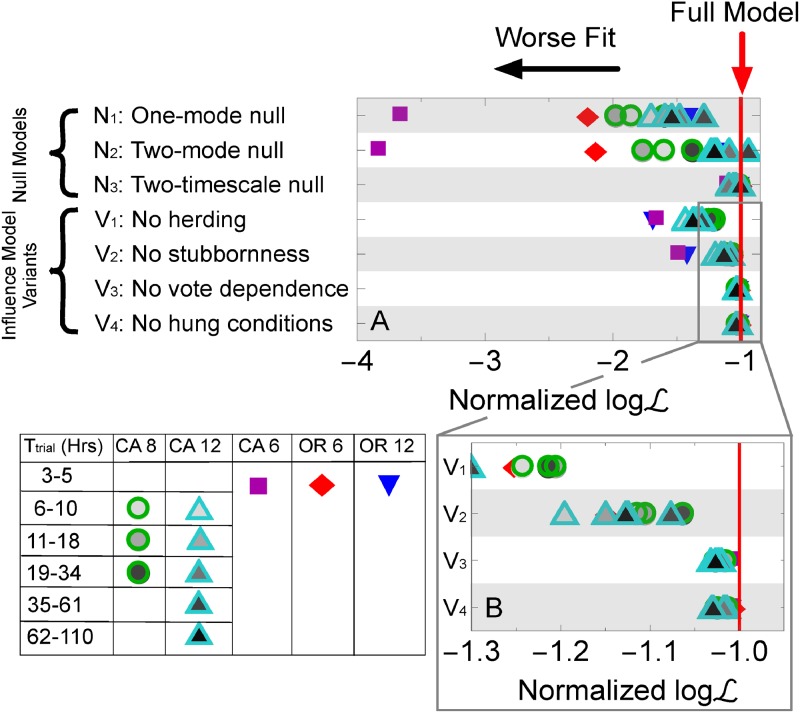
Comparing the influence with stubbornness model to null models. We compare the influence with stubbornness model fit to the one- and two-mode null models and the two-timescale model for the CA 12-juror data with *T*_trial_ equal to 6-8 hours. These figures serve as examples of the typical fit quality. (a) $\text{Pr}(V_{\text{p}}^{\text{f}}/N)$ versus $V_{\text{p}}^{\text{f}}/N$, (b) Pr(*T* ≥ *T*_delib_) versus *T*_delib_, and (c) 〈*T*_delib_〉 versus $V_{\text{p}}^{\text{f}}/N$.

To help answer this question, we created several models in which jurors made decisions independent of each other, but may plausibly create similar distributions to the ones we see. We first create an independent, random vote null model with which to compare other models. For this first model, for each dataset, we reshuffle all juror votes, which creates a binomial distribution of final votes. Not surprisingly, this “one-mode null model” fits data poorly; therefore we propose a slightly more nuanced “two-mode null model.” We split the jury data into those with majority for-plaintiff final votes ($V_{\text{p}}^{\text{f}}/N>0.5$) and the rest ($V_{\text{p}}^{\text{f}}/N\leq 0.5$), reshuffle juror votes of each subset separately, and then combine the distributions. In both cases, we fix $Pr(T_{\text{delib}}|V_{\text{p}}^{\text{f}}/N)$, the conditional probability for juries to stop deliberating at time *T*_delib_, given the fraction of for-plaintiff votes in the final vote, $V_{\text{p}}^{\text{f}}/N$, to exactly match the empirical data, as an unrealistic but best-case scenario of these null models. Both models produce poor fits of the data compared to other models (Figs 4e and 3 in the main text and Fig C in the S1 File), with the exception of CA 12 (*T*_trial_ = 34 − 61 hours) in which the two-mode model fits data better than any influence model tested. Overall, however, a simple model in which opinions are picked at random, independently of each other, does not provide a compelling explanation of the data. We also create a “two-timescale” null model of the deliberation time distribution, in which the time for each juror to make their pre-determined final decision is independent (exponentially distributed), but depends on whether their decision is for the plaintiff or not (hence “two-timescale”). Namely, we assume jurors stop at a rate *r*_pl_ if they eventually vote for the plaintiff or *r*_def_ otherwise. We converted these rates to the probability they stop per hour. Deliberation ends when the last juror makes their final decision. Separate fitting parameters are used for for-plaintiff and for-defendant votes because for-plaintiff votes usually take longer than for-defendant ones (p-value < 2 × 10^−2^ based on the Mann-Whitney U test for CA 6, CA12, OR 6, and OR 12 datasets, no significant difference for the CA 8 dataset), and it allows for this null model to better agree with the data. We determined distributions by Monte Carlo sampling 10^5^ times for each $V_{\text{p}}^{\text{f}}/N$ such that $Pr(V_{\text{p}}^{\text{f}}/N)$ is fixed to be the empirical data distribution as a best-case scenario. In this way, the two-timescale null model is meant to explain how juries stop deliberating, not how they reach their final vote. We find, however, that this model creates a poorer fit to the observed data than the influence with stubbornness model (to be discussed shortly), despite artificially fixing $Pr(V_{\text{p}}^{\text{f}}/N)$. While other plausible time distributions could be used and the assumption of a homogeneous distribution might not be ideal, disagreement between this idealized model and data point to limitations in similar null models.

### Findings

**Fig 5:**
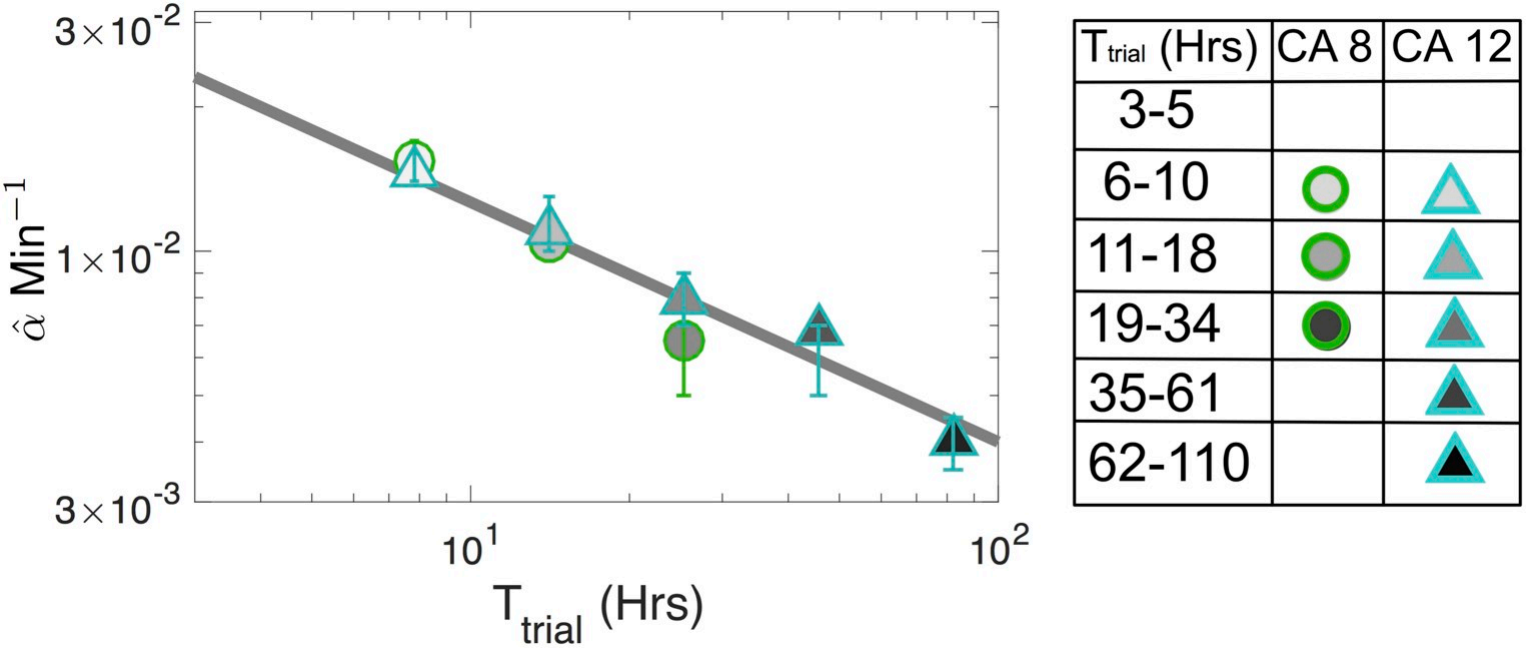
The scaling of the stopping rate versus the trial time. Solid line is *α* ∼ (*T*_trial_)^−1/2^, and error bars represent 90% confidence intervals in the mean.

What does the influence with stubbornness model suggest about jury deliberation? To begin to answer this question, we examine the best-fit model parameters for the different datasets (Tables A & B in the S1 File). Similar results are found when we look at criminal data from Oregon as well (Table C in the S1 File). First, we see that the fitted stubbornness rate is usually much lower when juries are hung ($\hat{f}<1$), which suggests that, under hung conditions, jurors significantly reduce the rate at which they stick to their current opinion. Also, the positive estimated values of $\hat{\alpha }$ indicate that juries are more likely to stop deliberating when they reach near-consensus. Further, $\hat{p}>0.5$ implies herding occurs within the jury, and $\hat{\mu }>0{\text{min}}^{-1}$ implies jurors keep their most recent opinion with increasing stubbornness. In Fig 5, we see that a parameter in the influence with stubbornness model, $\hat{\alpha }$, follows the power law relation $\hat{\alpha }∼{\left(T_{\text{trial}}\right)}^{-1/2}$, which agrees with Fig 1b because $T_{\text{delib}}∼{\hat{\alpha }}^{-1}$ (Eq (3)). We propose a possible mechanism for the scaling relationship *T*_delib_ ∼ (*T*_trial_)^1/2^: over the course of a trial, the amount of data juries will remember and deliberate on, *D*, might follow a random walk with a reflecting boundary condition at 0, which implies that ${\hat{\alpha }}^{-1}∼\langle T_{\text{delib}}\rangle ∼\langle D\rangle ∼{\left(T_{\text{trial}}\right)}^{1/2}$ (see Supporting Information). We also notice that, across all the data, the herding probability, $\hat{p}$, is highest when juries are smallest (Tables A, B, & C in the S1 File), while this value drops significantly for datasets with larger *N* (p-value < 0.003 between any *N* = 6 dataset and any *N* = 12 dataset). Previous studies on jury size found that larger juries become hung more frequently [56, Page 459], possibly because they have a minority opinion able to better resist the majority, and cite Asch’s “truthful partners” [34, 35] as a motivating reason. Our study provides evidence of this explanation because larger juries have smaller $\hat{p}$, and therefore jurors that are less likely to follow the majority opinion.

## Discussion

In the introduction, we laid out four motivations for the current research. First, we want to understand how groups make decisions that do not end in consensus. Second, we want to find ways empirical data can provide insight into the role influence plays in decision-making. Third, we aim to test the hypothesis that opinion formation is impacted by increasing stubbornness [5]. Finally, we want to determine whether we can use our modeling framework to better understand jury deliberation as a case study.

We create an influence model with increasing stubbornness that captures many properties seen in jury deliberation, including the distribution of final votes and deliberation times. This model, which intrinsically captures the lack of consensus in juries, is able to fit the data better than alternative models without influence or without stubbornness. Data is therefore in agreement with the hypothesis that both influence and stubbornness together affect decision making in juries. Increasing stubbornness was previously found to be important for explaining voting patterns in elections [5], which suggests that it may be an important mechanism in group decision-making. Despite the differences in data, the underlying mechanism is consistent. Furthermore, we find that deliberation times scale with the square root of trial times, which can be modeled as a random walk process, and that larger juries have less consensus, in agreement with theories of conformity [34, 35]. Both of these findings are captured by independent parameters of this model.

Future work is necessary to better understand how stubbornness and influence affects collective wisdom. For example, in a recent theoretical paper [36], correlations between jurors were found to reduce the accuracy of a decision, and sometimes create judgments with lower accuracy than individual jurors. In contrast, sequential voting, in which individuals base their decision on the popularity of decisions in the past, has been shown to significantly improve the wisdom of crowds [57, 58]. The benefit or risk of deliberation needs to be more thoroughly explored. We are also not aware of any paper that discusses how stubbornness can help or hurt decision quality, especially of jury decisions. Our work could also be extended by building more accurate models and better addressing data heterogeneity. Although the influence with stubbornness model is the best model that captures the data’s qualitative results, most of the data is statistically significantly different from the model, based on the two-dimensional Kolmogorov-Smirnov test (p-value < 0.1) [59], pointing to a need for more nuanced models to better explain the data. Another, more fundamental problem, however, in the datasets is heterogeneity: trials vary in complexity and jurors differ across trials, which can affect how decisions are reached. This may be addressed, however, with controlled experiments in which several groups separately deliberate on the same, or very similar, information. Data on how opinions change over time, as well as the time for juries to reach a verdict can provide tantalizing clues about the underlying mechanism of opinion dynamics.

## Supporting information

S1 File Supporting information: Evidence of herding and stubbornness in jury deliberations. To complement the main text, we discuss theory and data in more detail. First, we show why deliberation times increase in the hung-juror regime the closer the model is to consensus (see the deliberation time in Fig 4c of the main text). This also matches the findings we see in data (Fig 1a main text). Next, we show that criminal trial data show qualitatively similar findings to civil trial data we discuss in the main text. Findings therefore do not appear to be specific to civil cases alone. Finally, we discuss correlates of various attributes, which reveal assumptions we make in the main text are well-grounded.
(PDF)
