# Supplementary material for: Inferring models of opinion dynamics from aggregated jury data
Source: PLoS One. 2019 Jul 1;14(7):e0218312. doi: 10.1371/journal.pone.0218312 (PMC6602184; doi:10.1371/journal.pone.0218312)
Supplement: S1 File — To complement the main text, we discuss theory and data in more detail. First, we show why deliberation times increase in the hung-juror regime the closer the model is to consensus (see the deliberation time in Fig 4c of the main text). This also matches the findings we see in data (Fig 1a main text). Next, we show that criminal trial data show qualitatively similar findings to civil trial data we discuss in the main text. Findings therefore do not appear to be specific to civil cases alone. Finally, we discuss correlates of various attributes, which reveal assumptions we make in the main text are well-grounded. (PDF) [file pone.0218312.s001.pdf]

Supporting Information: Inferring Models of Opinion Dynamics from Aggregated Jury Data

ABSTRACT

1 Data Cleaning

See Table 2 in the main text for a summary of the amount of data before and after cleaning. For the CA dataset, we only include datapoints where we have trial time, deliberation time in hours, final vote, and one count to deliberate on. Once cleaned, we have 53 trials for CA 6, 338 trials for CA 8, and 1726 trials for CA 12 out of 6482 total trials. We do not know whether the kept data suffers from selection bias after removing data that is incomplete. Data may be incomplete, for example, for trials that take an especially short time or for hung juries because jurors may have less motivation to fill out long forms describing trials and deliberations if the trial seems inconsequential. The qualitative similarities of datasets, however, despite the varying amount of data removed, suggest that any bias should not significantly affect our results (as shown in Fig. 1 in the main text). In the OR dataset, we removed all data where we did not simultaneously know deliberation time and final vote. This, however, only removes 4 trials; once cleaned, there were 207 trials for OR 6 jury data, and 951 trials for OR 12 jury data. Finally, for the WA and NE datasets, we removed data where we did not simultaneously know both the trial time and deliberation time.

In addition, we rescale the CA data trial time when we create Figs 1, 3, and 5 in the main text. The CA data only recorded trial time in days. In Fig 1b and subsequent plots, we want to plot all data with a standard unit of time (hours). We notice, however, that there are approximately 4.5 trial hours for each recorded trial day in the NE and WA data. We therefore made a simple assumption for the CA data that each trial day consisted of 4 trial hours. This does not affect model fits, and any conversion we make would only shift the CA plot markers in Fig. 1b along the x-axis; this does not change the scaling law.

2 How Deliberation Time is Affected by the Final Vote

It might not be intuitive why the deliberation time, T_{delib}, is highest when jurors are near consensus in both the data and the influence model, while T_{delib} is lower when jurors are evenly split (see Figs. 1 & 2 in the main text). In this section, we present a simple Markov chain model to better understand this finding. We find that the fraction of jurors voting for the plaintiff, $V_{\text{p}}^{\text{f}}/N = 0.5$ is rare, as seen in Fig. 2a in the main text. This finding is likely related to the influence model becoming the Majority Voter Model (MVM) when $\alpha = \mu = 0$, i.e., when juries do not stop deliberating and there is no jury stubbornness, because $V_{\text{p}}(t)/N = 1/2$ is known to be unstable past a critical point in the MVM when the influence of neighbors changes from weak (and opinions are evenly split) to strong (and there is near-unanimous agreement)[1,2]. Using this numerical finding, we create a similar, but much simpler, model in which the number of jurors voting for the plaintiff is represented as a node in a Markov chain, and there is a bias for juries to have greater agreement (see Fig. S1). In the model, juries begin evenly split ($V_{\text{p}}(0)/N = 1/2$) but can transition to a new state, $V_{\text{p}}(1)/N \pm 1/N$, with probability $(1-s')/2$. Once jurors reach this new state, they can achieve greater consensus, $V_{\text{p}}(1)/N \pm 2/N$, with probability $(1-s')/2$, or stay in the current state. This pattern can

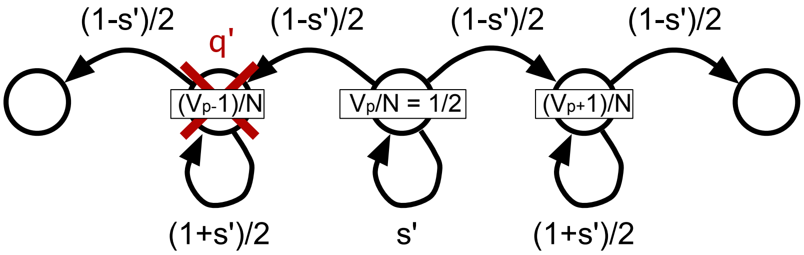

Figure A. A Markov model approximation of the influence model. We assume states change as a Markov chain, therefore with probability s' we remain in state $V_{\text{p}}(t)/N = 1/2$, but transition to $V_{\text{p}}(t)/N = 1/2 \pm 1/N$ with probability $(1-s')/2$. Once we are at a new state, we either transition to $V_{\text{p}}(t)/N = 1/2 \pm 2/N$ with the same probability or stay in the current state. Finally, with probability q', juries stop deliberating.

continue until juries stop deliberating with probability q' at each timestep. Recall that, in the influence model seen in the main text, a juror will choose not to re-evaluate their opinion with probability s, and even if they do re-evaluate, they may choose to keep their original opinion, therefore it is reasonable for self-loops to exist in the Markov chain model. That said, because s is often less than 1, and $p > 1/2$, it is reasonable to assume that opinions develop stronger pluralities over time, ergo the Markov chain model captures many qualitative features of the influence model. Starting from time $t = 1$, we find that the probability a jury is evenly split by the time they stop deliberating at time t is

$$Pr(V_{\mathrm{p}}^{\mathrm{f}}/N = 1/2, t) = q'(1-q')^{t-1}(s')^{t} \tag{1}$$

which implies that

$$Pr(1/2) = \frac{q's'}{1-s'(1-q')} \tag{2}$$

and the probability the jury stops deliberating with an opinion $V_{\mathrm{p}}^{\mathrm{f}}/N = 1/2 \pm 1/N$ at time t is

$$Pr(V_{\mathrm{p}}^{\mathrm{f}}/N = 1/2+1/N, t) = Pr(1/2-1/N, t) = q'(1-q')^{t-1}\left\{\left[\frac{1+s'}{2}\right]^{t-1} - (s')^{t-1}\right\}, \tag{3}$$

and the probability over all time is

$$Pr(1/2 \pm 1/N) = \frac{q'(1-s')}{[(q'-1)s'+1][(q'-1)s'+q'+1]}, \tag{4}$$

Using $Pr(1/2, t)$ and $Pr(1/2 \pm 1/N, t)$, we can also find the mean deliberation time conditioned on the final vote:

$$\langle T_{\mathrm{delib}}(1/2)\rangle = \frac{s'q'}{[1-s'(1-q')]^2} \tag{5}$$

and

$$\langle T_{\mathrm{delib}}(1/2 \pm 1/N)\rangle = \frac{q'\left\{(q'-1)^2 s'^3 - [(q'-2)q'+3]s' + 2\right\}}{[(q'-1)s'+1]^2[(q'-1)s'+q'+1]^2}, \tag{6}$$

where $\langle . \rangle$ is the average. If $s' \to 0$ (in other words, $V_{\mathrm{p}}(t)/N = 1/2$ is very unstable), then we find that

$$Pr(1/2) = s'q', \tag{7}$$

and

$$Pr(1/2 \pm 1/N) = \frac{q'}{q'+1}, \tag{8}$$

In comparison

$$\langle T_{\mathrm{delib}}(1/2)\rangle = q's', \tag{9}$$

and

$$\langle T_{\mathrm{delib}}(1/2 \pm 1/N)\rangle = \frac{2q'}{(q'+1)^2}. \tag{10}$$

The probability that deliberation stops at $V_{\mathrm{p}}^{\mathrm{f}}/N = 1/2$ is small, but so is the time that this deliberation would subsequently take. In comparison, $V_{\mathrm{p}}^{\mathrm{f}}/N = 1/2 \pm 1/N$ is more likely, but mean deliberation time is subsequently higher. If we continue to $V_{\mathrm{p}}^{\mathrm{f}}/N = 1/2 \pm 2/N$, T_{delib} is expected to further increase because it takes at a minimum number of timesteps to reach the state. In short, the Markov chain model helps explain why T_{delib} is low when the jury is evenly split, even though the probability for a jury to be evenly split is low as well. Furthermore, the Markov chain model helps explain why deliberation increases with greater consensus, at least until $V_{\mathrm{p}}^{\mathrm{f}}/N \approx 0.3$ and $V_{\mathrm{p}}^{\mathrm{f}}/N \approx 0.6$, when quitting rates substantially increase in the influence model, therefore lowering T_{delib} again.

Random Walk Stopping Rate

We notice in Fig. 1b of the main text that the deliberation time scales as the square root of the trial time. In this section, we derive a mechanistic explanation of this scaling law. If we assume that the amount of information jurors accumulate is D, which we assume follows a random walk with a reflective boundary at $D = 0$ (people cannot have negative information), and the amount of time users deliberate scales as $T_{\text{delib}} \sim D$, then $\alpha^{-1} \sim D$, where α is proportional to the quitting rate in the influence model. To better understand how D affects the dynamics, recall that

$$Pr(D|T) = \binom{T+1}{(T+D-1)/2} \frac{D}{T+1}, \tag{11}$$

where T is the number of timesteps. Taking T to be large, using the Sterling's formula, and dropping non-leading terms,

$$Pr(D|T) \approx \frac{2D}{T} e^{-(D^2/T)}. \tag{12}$$

This immediately implies that $\langle D \rangle \sim T^{1/2}$. T is not, as of yet, explicitly defined because T is still the number of timesteps and not an actual time. We can, however, set $T \sim T_{\text{trial}}$, and, because $T_{\text{delib}} \sim D$, $T_{\text{delib}} \sim T^{1/2} \sim T_{\text{trial}}^{1/2}$, in agreement with what we find empirically, therefore $\hat{\alpha} \sim T_{\text{trial}}^{-1/2}$.

3 Data Details and Model Fits

First, we show, in detail, the amount of data modeled after splitting by state and trial time. Next, we discuss variants of the influence model described in the paper. We then discuss criminal data modeled in the OR dataset, before we describe feature correlations.

Table A. Data Descriptions

Data Description		
Data	T_{trial} (Hrs)	Num. Trials
OR 6	–	101
OR 12	–	560
CA 6	–	53
CA 8	6-10	171
CA 8	11-18	121
CA 8	19-34	32
CA 12	6-10	502
CA 12	11-18	656
CA 12	19-34	402
CA 12	35-61	111
CA 12	62-110	42

Table S1 details the amount of data for the OR and CA civil data split by trial time. As we describe in the beginning of the SI, the trial time is recorded in days, and the conversion from trial time hours to days is 4 hours per day.

3.1 Alternative Jury Models

We mention in the main text that removing all hung conditions in the herding and stubbornness model will produce a poorer fit (see Fig. 4 in the main text and Fig. S2). To better understand why this is the case, we separately remove the dependence of the

Table B. Model Parameter Fits

Parameter Fits								
Data	$\hat{p}$	90% Conf.	$\hat{\alpha}$ (min^{-1})	90% Conf.	$\hat{\mu}$ (min^{-1})	90% Conf.	$\hat{f}$	90% Conf.
OR 6	0.956	0.94-0.98	0.0163	0.014-0.020	0.17	0.09-0.33	0.037	0.0-0.08
OR 12	0.873	0.85-0.89	0.012	0.010-0.014	0.070	0.05-0.09	0.028	0.0-0.09
CA 6	0.975	0.97-0.98	0.0080	0.007-0.009	0.042	0.03-0.07	1.0	0.9-1.0
CA 8	0.913	0.85-0.95	0.0154	0.014-0.017	0.37	0.2-0.7	0.11	0.0-0.2
CA 8	0.858	0.80-1.0	0.0104	0.010-0.012	0.49	0.2-1.0	0.24	0.0-0.6
CA 8	0.775	0.75-0.80	0.0065	0.005-0.008	0.50	0.40-0.67	0.011	0.0-0.02
CA 12	0.864	0.83-0.91	0.0146	0.014-0.016	0.63	0.4-1.0	0.22	0.1-0.4
CA 12	0.811	0.78-0.86	0.011	0.010-0.013	0.38	0.2-0.5	0.25	0.2-0.4
CA 12	0.812	0.80-0.85	0.0079	0.007-0.009	0.51	0.4-0.6	0.19	0.1-0.4
CA 12	0.747	0.71-0.80	0.0068	0.005-0.007	0.28	0.15-0.67	0.13	0.1-0.2
CA 12	0.765	0.75-0.78	0.0040	0.0035-0.0045	0.33	0.2-1.0	0.001	0.0-0.015

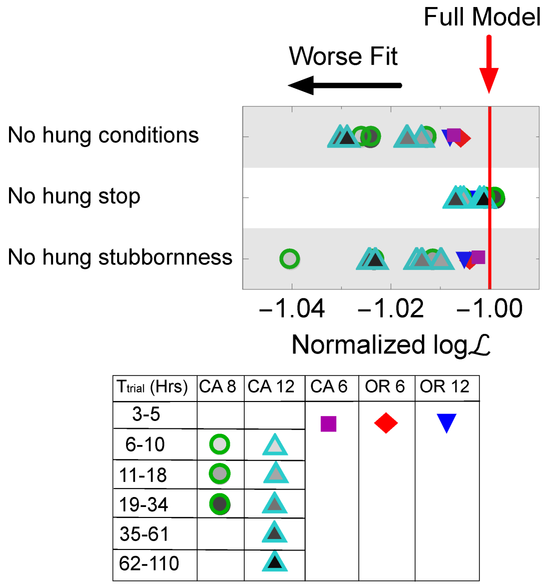

Figure B. A comparison between the normalized log-likelihood of three model variants for the CA 6, CA 8, CA 12, OR 6 and OR 12 datasets: no hung conditions, in which the influence model does not change when juries hang, no hung stop, in which the rate juries hand depends on the current vote but not whether the jury is hung, and finally no hung stubbornness, in which the stubbornness rate does not depend on whether the jury is hung. In this plot, a value of -1 corresponds to the fit of the full model. Values less than -1 correspond to fits worse than the full model.

Table C. Model Parameter Fits: OR 6 & OR 12 Criminal Cases

Parameter Fits										
Data	T_{trial} (Hrs)	Num. Trials	$\hat{p}$	90% Conf.	$\hat{\alpha}$ (min^{-1})	90% Conf.	$\hat{\mu}$ (min^{-1})	90% Conf.	$\hat{f}$	90% Conf.
OR 6 Crim.	–	104	0.961	0.92-1.0	0.0181	0.016-0.022	0.36	0.25-0.50	0.045	0.0-0.1
OR 12 Crim.	–	374	0.904	0.89-0.91	0.0116	0.010-0.014	0.018	0.01-0.05	0.32	0.0-0.6

quitting rate, q, and stubbornness rate, $\mu_{\text{eff}}(t)$, on whether the jury is hung. In the former case, we see a small change in the log-likelihood, but in the latter case, the log-likelihood has a more significant drop. This suggests that jurors depend more on changing their stubbornness rate than changing their quitting rate when they avoid hanging.

3.2 Oregon Criminal Cases

In this section, we compare data and fits for criminal and civil cases in Oregon. The reason we separate the data is both because the requirements for a verdict are different (ten out of twelve jurors are need to agree instead of nine out of twelve, although five out of six still need to agree in six-person juries), and the motivations for reaching a decision may be different. Overall, we find quantitatively similar findings between criminal and civil cases. First, we compare OR 6 and OR 12 attributes seen in Fig. 2 of the main text (Fig. S3). We find that $\langle T_{\text{delib}} \rangle$ is higher for OR 6 criminal cases compared to civil cases, but the trend is not as clear for the OR 12 cases (Fig. S3a). That said, in all cases we see that $\langle T_{\text{delib}} \rangle$ is higher when there is greater disagreement among jurors. We also find that juries are commonly found to reach a verdict and hung juries are rare (Fig. S3b). Finally, we see that $Pr(T_{\text{delib}})$ is almost exactly the same for both civil and criminal cases.

Next we compare the fits for civil and criminal cases. Overall, we find that civil and criminal cases fit each model similarly well (Fig. S4). For example, the one-mode and two-mode null models give some of the worst fits, and the two-timescale model was the best null model, although it was still worse than the full influence model. Furthermore, removing either herding or stubbornness from the full influence model produces a much worse fit, while removing the vote dependence or hung conditions has a much smaller effect. We also see the same qualitative trends when we separately remove the dependence of the stubbornness rate or quitting rate on whether the jury is hung (Fig. S5). In both the criminal and civil cases, removing the stubbornness rate's dependence on whether a jury is hung creates a significantly worse fit compared to removing the quitting rate's dependence.

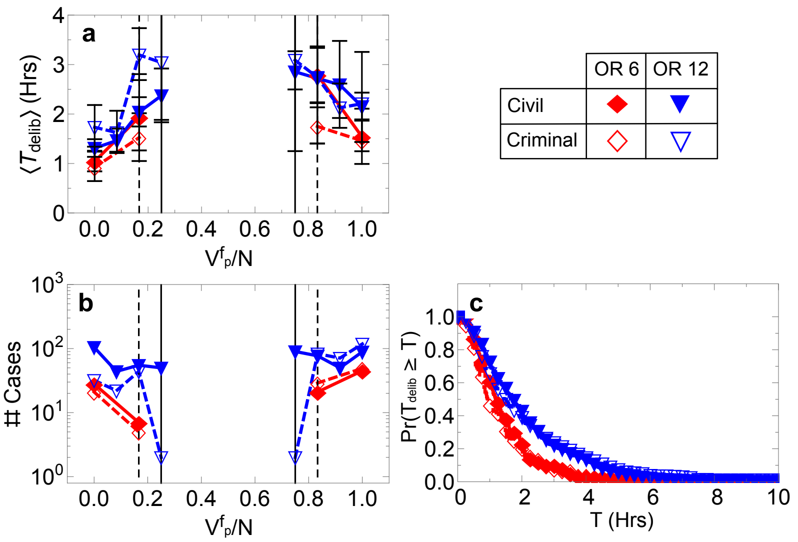

Figure C. OR 6 and OR 12 Data attributes for Criminal and Civil Cases. (a) The mean deliberation versus V_p^f/N, the fraction of jurors voting for the plaintiff (or voting guilty in criminal cases). (b) The distribution of V_p^f/N, (c) The complementary cumulative distribution of deliberation times, T_{delib}. Data is taken from[3] and error bars represent 90% confidence intervals in the mean.

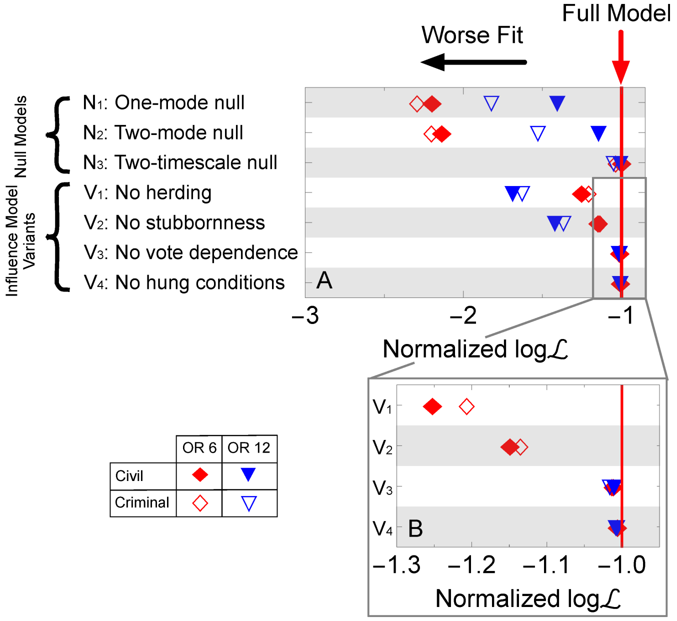

Figure D. Comparison of models for OR 6 and OR 12 civil and criminal cases. (a) The normalized log-likelihood of the one-mode, two-mode, and two-timescale null models, along with the "no herding" model, "no stubbornness" model, "no vote dependence" model, and the "no hung conditions" model (see main text for definitions). (b) In a zoomed-in graph, the influence model variants seen in (a) perform worse than the full influence model. Overall, all models perform worse than the full model.

3.3 Correlations Between Jury Attributes

In this section, we discuss correlations between various attributes, in order to better understand how to model jury dynamics. First, we look at how the jury size affects the deliberation time (Fig. S6), and notice very little correlation between the two. This contrasts with many models of opinion dynamics in which deliberation time strongly correlates with system size[4,5]. Next, we compare how the trial time depends on the final vote (Fig. S7). Interestingly, although both the trial time and the final vote strongly affect the deliberation time (Fig. 2 in the main text), neither are strongly correlated with each other. We use this

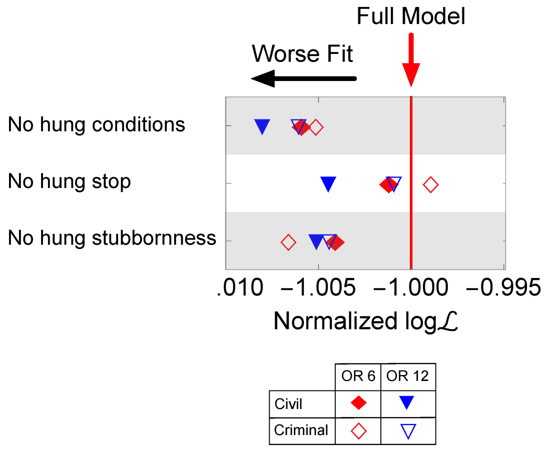

Figure E. A comparison of normalized log-likelihood values for three model variants of OR 6 and OR 12 civil and criminal data: no hung conditions, in which the influence model does not change when juries hang, no hung stop, in which the rate juries hand depends on the current vote but not whether the jury is hung, and finally no hung stubbornness, in which the stubbornness rate does not depend on whether the jury is hung. In this plot, a value of -1 corresponds to the fit of the full model. Values less than -1 correspond to fits worse than the full model. Overall, all the models perform worse than the full influence model. Compare to Fig. B.

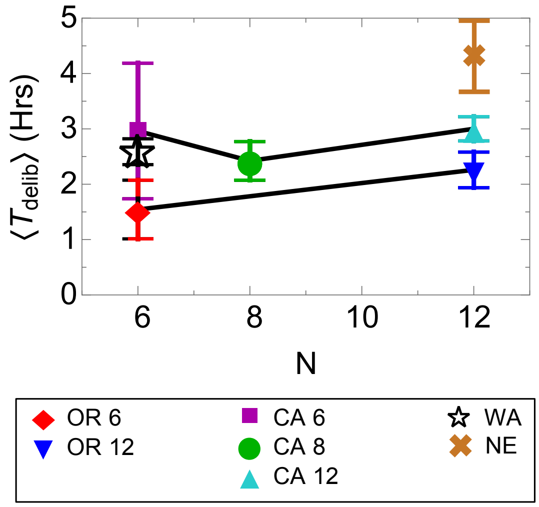

Figure F. The mean deliberation time, $\langle T_{\text{delib}} \rangle$, versus number of jurors, N, for all the data sets. Overall, there is little dependence between $\langle T_{\text{delib}} \rangle$ and N. Error bars represent 90% confidence intervals in the mean (via bootstrapping).

property to find separate mechanisms for the correlation between each attribute and deliberation time.

Finally, we plot the probability a typical voter will vote for the plaintiff (or vote guilty in criminal cases), Pr(Outlier For Plaintiff), versus the vote of all the other jurors for OR 12 (Fig. S8). We find a strong correlation between the two in civil cases and criminal cases, therefore juror opinions are not independent, which gives strong evidence that herding may exist in juries. We find Pr(Outlier For Plaintiff) by determining how many trials end with verdict $V_{\text{p}}^{\text{f}} = V_{\text{p,N-1}}^{\text{f}} + 1$, corresponding to the outlier juror voting for the plaintiff, and how many trials end with $V_{\text{p}}^{\text{f}} = V_{\text{p,N-1}}^{\text{f}}$, corresponding to the outlier juror voting for the defendant. The probability, Pr(Outlier For Plaintiff), is simply

$$Pr(\text{Outlier For Plaintiff}) = \frac{Pr\left(V_{\text{p},N-1}^{\text{f}} + 1\right)}{Pr\left(V_{\text{p},N-1}^{\text{f}} + 1\right) + Pr\left(V_{\text{p},N-1}^{\text{f}}\right)}. \tag{13}$$

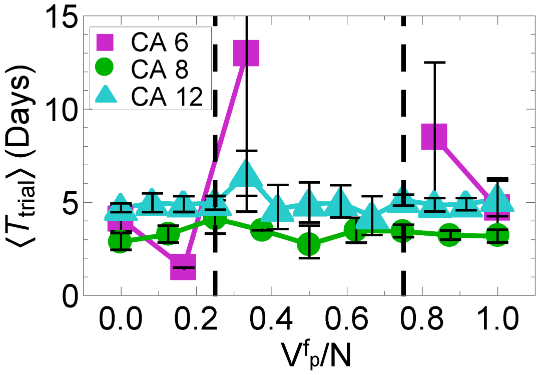

Figure G. The mean trial time, $\langle T_{\mathrm{trial}} \rangle$, versus the final fraction of jurors voting for the plaintiff, $V_{\mathrm{p}}^{\mathrm{f}}$, for CA 6, CA 8, and CA 12. Error bars represent 90% confidence intervals in the mean (via bootstrapping). Overall, there is little dependence between $\langle T_{\mathrm{trial}} \rangle$ ad $V_{\mathrm{p}}^{\mathrm{f}}$, although both strongly affect $\langle T_{\mathrm{delib}} \rangle$, the mean deliberation time.

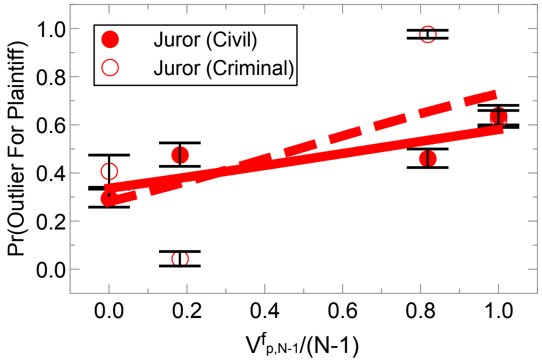

Figure H. Correlations between juror votes for criminal and civil cases in OR 12. The y-axis is the probability a random juror, which we call the outlier, votes for the plaintiff, while the x-axis is the fraction of all the other jurors voting. We find that there is a correlation between jurors: the fraction of jurors voting for the plaintiff correlates with the likelihood that the removed juror also votes for the plaintiff. This is further demonstrated in the logistic regressions, $y = \frac{1}{1+e^{-(\beta_0+\beta_1 x)}}$, with coefficient $\beta_1 = 1.0$ (95% confidence [0.6-1.4]) for civil cases (solid red line), and $\beta_1 = 1.9$ (95% confidence [1.4-2.5]) for criminal cases (dashed red line). Error bars are standard errors.

References

1. de Oliveira, M. J. Isotropic majority-vote model on a square lattice. *Journal of Statistical Physics* **66**, 273–281 (1992).
2. Liggett, T. *Interacting Particle Systems* (Springer-Verlag, New York, NY, 1985).
3. Grofman, B. *Multnomah County [Oregon] Jury Project, 1973-1976.* (Inter-university Consortium for Political and Social Research, Ann Arbor, MI, 1984). URL http://doi.org/10.3886/ICPSR09030.v1.
4. Sood, V., Antal, T. & Redner, S. Voter models on heterogeneous networks. *Phys. Rev. E* **77**, 041121 (2008).
5. Krapivsky, P. L. & Redner, S. Dynamics of majority rule in two-state interacting spin systems. *Phys. Rev. Lett.* **90**, 238701 (2003).
